# Mapping area of habitat for inland wetland species

**DOI:** 10.1111/cobi.70163

**Published:** 2025-10-29

**Authors:** Francesca A. Ridley, Catherine A. Sayer, Louise Mair, Daniele Baisero, Monika Böhm, Thomas M. Brooks, Stuart H. M. Butchart, Viola Clausnitzer, Jeorg Freyhof, Günther Grill, Ian Harrison, Randall R. Jiménez, Bernhard Lehner, Nicholas B. W. Macfarlane, Andrew J. Plumptre, Arnout van Soesbergen, Thomas A. Worthington, Thomas Starnes

**Affiliations:** ^1^ School of Natural and Environmental Sciences Newcastle University Newcastle upon Tyne UK; ^2^ IUCN SSC Freshwater Conservation Committee IUCN Species Survival Commission Gland Switzerland; ^3^ IUCN SSC Global Biodiversity Framework Task Force IUCN Species Survival Commission Gland Switzerland; ^4^ IUCN Cambridge UK; ^5^ KBA Secretariat c/o BirdLife International Cambridge UK; ^6^ BirdLife International Cambridge UK; ^7^ Global Center for Species Survival Indianapolis Zoo Indianapolis Indiana USA; ^8^ IUCN Gland Switzerland; ^9^ World Agroforestry Center (ICRAF) University of the Philippines Los Baños Los Baños Philippines; ^10^ Institute for Marine and Antarctic Studies University of Tasmania Hobart Tasmania Australia; ^11^ Department of Zoology University of Cambridge Cambridge UK; ^12^ Senckenberg Research Institute Görlitz Germany; ^13^ Museum für Naturkunde Leibniz Institute for Evolution and Biodiversity Science Berlin Germany; ^14^ Confluvio Consulting Montreal Quebec Canada; ^15^ IUCN San José Costa Rica; ^16^ Department of Geography McGill University Montreal Quebec Canada; ^17^ IUCN Washington, DC USA; ^18^ UN Environment Programme World Conservation Monitoring Centre Cambridge UK; ^19^ Department of Geography King's College London London UK

**Keywords:** aquatic conservation, freshwater, habitat suitability modeling, inland waters, IUCN Red List, spatial ecology, agua dulce, aguas continentales, conservación acuática, ecología espacial, Lista Roja de la UICN, modelos de idoneidad de hábitat

## Abstract

Area of habitat (AOH) maps provide a high‐resolution representation of the habitat available in a species’ range and can support conservation policy and planning processes. However, until recently, there was insufficient knowledge on the distribution of inland wetlands and freshwater biodiversity to develop AOH mapping methods specifically tailored to inland wetlands. We used a combined empirical and thematic approach to translate inland wetland habitat classes in the International Union for Conservation of Nature (IUCN) Habitats Classification Scheme into spatially explicit wetland‐cover types derived from the Global Lakes and Wetlands Database 2 and the World Karst Aquifer Map. The AOH was subsequently estimated as the area in the mapped range that corresponded to each species’ habitat and elevation associations. We developed and tested the method with IUCN Red List assessment data, range maps, and point locality data for fishes, odonates, decapod crustaceans, and mollusks (22,876 species). The method performed well in comparison with similar methods already developed for terrestrial mammals, birds, amphibians, and reptiles. The mean map prevalence (proportion of area in a species’ range that was AOH) was 18–32% for each taxonomic group. Based on data on known localities of occurrence, 78–100% of species per taxonomic group had a higher classification accuracy than expected if AOH were distributed in the range at random. This represents an increased accuracy in the distribution of wetland species. Our study represents the first attempt to distinguish between inland wetland habitat subclasses and to include subterranean habitats in an AOH mapping method. Our method will facilitate the inclusion of previously underrepresented taxa in key conservation tools and analyses and is expected to increase the accuracy of AOH mapping for any species associated with inland wetlands.

## Abstract (es)

Mapeo del área de hábitat de las especies de humedales continentales

**Resumen:**

Los mapas del área de hábitat (AH) proporcionan una representación de alta resolución del hábitat disponible en el área de distribución de una especie y pueden servir de apoyo a las políticas de conservación y los procesos de planificación. Sin embargo, hasta hace poco, no se disponía de conocimientos suficientes sobre la distribución de los humedales continentales y la biodiversidad de agua dulce para desarrollar métodos de mapeo del AH específicamente adaptados a los humedales continentales. Utilizamos un enfoque empírico y temático combinado para traducir las clases de hábitats de humedales continentales del Esquema de Clasificación de Hábitats de la Unión Internacional para la Conservación de la Naturaleza (UICN) en tipos de cobertura de humedales espacialmente explícitos derivados de la Base de Datos Global de Lagos y Humedales 2 y del Mapa Mundial de Acuíferos Cársticos. Posteriormente, estimamos el AH como el área de la zona de distribución cartografiada que correspondía a las asociaciones de hábitat y elevación de cada especie. Desarrollamos y probamos el método con datos de evaluación de la Lista Roja de la UICN, mapas de distribución y datos de localización puntual de peces, odonatos, crustáceos decápodos y moluscos (22,876 especies). El método funcionó bien en comparación con métodos similares ya desarrollados para mamíferos terrestres, aves, anfibios y reptiles. La prevalencia media en el mapa (proporción del área en la zona de distribución de una especie que era AH) fue del 18‐32% para cada grupo taxonómico. Según los datos sobre las localidades de ocurrencia conocidas, entre el 78% y el 100% de las especies por grupo taxonómico tuvieron una precisión de clasificación mayor de lo esperado si las AH se distribuyeran aleatoriamente en la zona de distribución. Esto representa una mayor precisión en la distribución de las especies de humedales. Nuestro estudio representa el primer intento de distinguir entre subclases de hábitats de humedales continentales e incluir hábitats subterráneos en un método de mapeo del AH. Nuestro método facilitará la inclusión de taxones anteriormente infrarrepresentados en herramientas y análisis clave para la conservación, y se espera que aumente la precisión del mapeo del AH para cualquier especie asociada a los humedales continentales.

## INTRODUCTION

Estimates of area of habitat (AOH) can support assessments of species’ extinction risk (Brooks et al., 2019), identification of key biodiversity areas (IUCN, 2016), and derivation of metrics for measuring progress in delivering science‐based targets for conservation action, such as the species threat abatement and restoration (STAR) metric (Mair et al., 2021). Despite inland wetland systems being highly threatened (Gardner & Finlayson, 2018; Grill et al., 2019) and of high ecological (Strayer & Dudgeon, 2010) and human value (Kaval, 2019; Lynch et al., 2023; WWF, 2022), to date there has been no attempt to map AOH in a way that is specifically targeted at inland wetland habitats. Existing terrestrial AOH mapping methods included inland wetlands but considered all inland wetland types as a single class. This method had lower classification accuracy and higher omission errors for wetlands than other land‐cover classes (Lumbierres, Dahal, Di Marco, et al., 2022).

A species’ AOH is defined as the habitat available to a species (i.e., habitat in its geographic range) (Brooks et al., 2019). The IUCN's Red List range maps represent the distributional limits of assessed taxa and hence contain areas from which the species is absent, due to lack of habitat or elevation or extirpation, for example, through overexploitation. The IUCN Red List range maps attempt to minimize omission errors but contain local commission errors (false presence) (IUCN, 2021). The IUCN Red List range maps for most freshwater species are mapped to river catchments and lakes (IUCN, 2021), delineated using HydroBASINS, a globally standardized hydrological framework (Lehner & Grill, 2013). Thus, AOH reduces commission errors by excluding unsuitable elevations and areas as defined in IUCN Red List assessments. Area of habitat mapping methods have been developed for terrestrial mammals, birds, amphibians, and reptiles (Beresford et al., 2011; Buchanan et al., 2011; Ficetola et al., 2015; Lumbierres, Dahal, Di Marco, et al., 2022; Nania et al., 2022; Rondinini et al., 2011) and comprehensively assessed marine taxa (Turner et al., 2024).

Mapping AOH for inland wetland habitats presents several challenges. First, many wetland‐dependent species rely on linear features, such as rivers and streams. The land‐cover classes surrounding such features dominate in raster datasets where each cell is a single class. For example, where a stream runs through a forest, it is the forest that is represented in the land‐cover dataset. Existing terrestrial AOH mapping methods typically are based on such datasets to represent the geographic occurrence of all inland habitat types (Lumbierres, Dahal, Soria, et al., 2022). Therefore, a direct application of existing AOH methods to wetland‐dependent species risks underrepresenting important habitat for many species.

Second, some species rely on seasonal habitats, such as temporary rain‐fed puddles of a few centimeters in depth that form in a variety of natural and human‐modified land‐cover types, including on rock surfaces or by roadsides (van Benthem Jutting, 1959). As such, the locations of such seasonal habitats vary. For example, the Neotropical dragonfly *Micrathyria ringueleti*, which is exclusively associated with small seasonal pools, has never been observed in the same place twice (Lozano & Muzón, 2017). This makes the geographic occurrence of these habitats difficult to predict and makes a methodology that is reproducible across a large number of species with high taxonomic and ecological variability hard to define.

Third, the challenge of data availability expressed for terrestrial vertebrates (Rondinini et al., 2011) is exacerbated in inland wetland species. AOH validation requires species’ occurrence data, which is particularly sparse for freshwater invertebrates (Troudet et al., 2017). Moreover, elevation data are often missing in IUCN assessments of species associated with freshwater systems (97% of mollusks, 86% of fishes, 85% of decapod crustaceans, and 51% of odonates are missing at least one elevation limit). Though multiple global datasets exist for terrestrial elevation and marine bathymetry, the bathymetry of inland waters has not been mapped globally.

There has, however, been a considerable increase in knowledge on the global distribution of inland wetland habitats and associated species over the last 20 years (McManamay et al., 2018; Strayer & Dudgeon, 2010), which, combined with recent IUCN assessments (Sayer et al., 2025), now makes global AOH mapping of freshwater species feasible for the first time. Multiple datasets on a range of lentic (still) and lotic (flowing) habitat types have been consolidated into a single database, the Global Lakes and Wetlands Database 2 (GLWD) (Lehner & Döll, 2004; Lehner et al., 2025). This database contains a composite raster map of wetland classes at 500‐m resolution and additional information on the fractional coverage of each class in each cell. Advances in the quantification of groundwater resources (Chen et al., 2017) present the opportunity to include subterranean habitats in global AOH mapping. Utilizing new, inland wetland‐specific datasets therefore has the potential to increase the accuracy of AOH maps for any species associated with inland wetland habitats.

We devised a reproducible and data‐driven method for mapping AOH for species associated with inland wetland habitats that includes the translation of IUCN inland wetland habitat classes into geographic wetland cover. The method was developed and tested using 22,876 species of fishes, odonates, decapod crustaceans (crayfishes, crabs, and shrimps), and mollusks associated with one or more inland wetland habitats in the IUCN Habitats Classification Scheme (IUCN, 2012). These taxonomic groups were chosen because they rely on inland wetland habitats for all, or part, of their life cycle and are not represented in applications of existing AOH mapping methodologies. Our aim was to develop, implement, and validate an accurate AOH mapping method that reduces the commission errors associated with species’ range maps while minimizing the introduction of omission errors and is reproducible across different taxonomic groups to improve thematic precision in mapping of AOH for inland wetland habitat types and to map AOH for many wetland‐dependent taxa.

## METHODS

**FIGURE 1:**
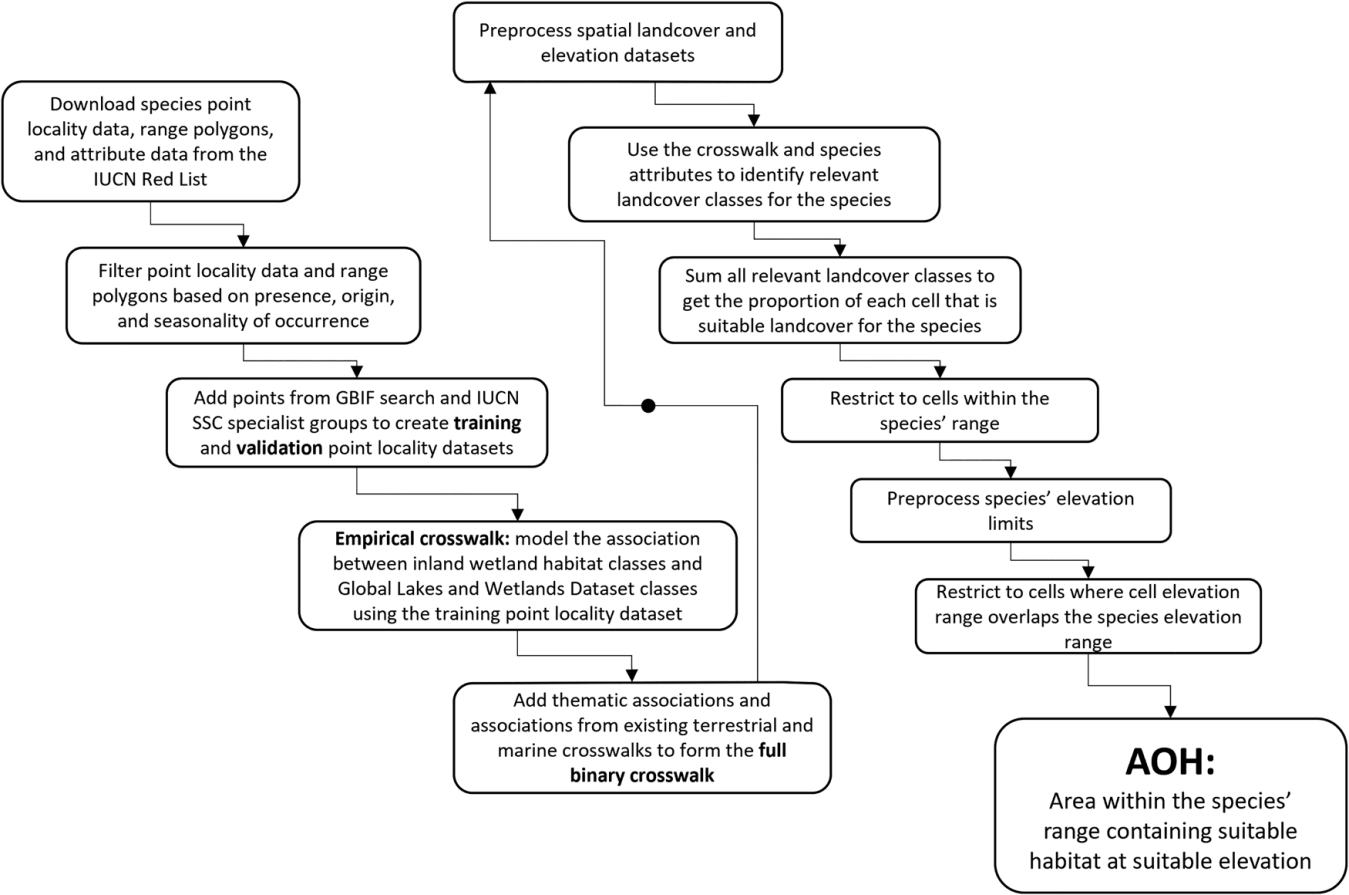
Step‐by‐step process for translating International Union for Conservation of Nature (IUCN) inland wetland habitat classes into mapped wetland cover and mapping area of habitat (AOH) for inland wetland species.

We defined AOH as the area in the species’ range that contains habitat at a suitable elevation. First, logistic regression models were used to translate inland wetland habitats (class 5 in the IUCN Habitats Classification Scheme) (Table 1) into wetland‐cover classes (Figure 1). The model output was combined with additional thematic associations to produce a crosswalk relating each inland wetland habitat to at least one relevant wetland class (Figure 1). Second, habitat for species was estimated by cross‐referencing species’ habitat and elevation associations from IUCN Red List assessments with the crosswalk and spatial datasets on wetland cover (a beta version of GLWD [Lehner et al., 2025] and WoKAM [Chen et al., 2017]) and elevation (forest and buildings removed digital elevation model [FABDEM] [Hawker et al., 2022]). The resulting AOH maps indicated the proportion of each raster cell that contained suitable habitat at a suitable elevation for each species. Finally, the maps were validated in a 2‐stage process that did not strictly rely on point locality data.

**TABLE 1:** Number of species used to develop the methodology associated with each inland wetland habitat class on the International Union for Conservation of Nature (IUCN) Red List.

Code	IUCN habitat class	Fish	Odonate	Decapod	Mollusk	Total
5.1	Permanent rivers, streams, and creeks (includes waterfalls)	10,610	4159	1989	1721	18,479
5.2	Seasonal, intermittent or irregular rivers, streams, and creeks	2033	278	73	115	2499
5.3	Shrub‐dominated wetlands	179	172	20	43	414
5.4	Bogs, marshes, swamps, fens, peatlands	1168	852	143	187	2350
5.5	Permanent freshwater lakes (>8 ha)	3159	577	208	846	4790
5.6	Seasonal or intermittent freshwater lakes (>8 ha)	728	183	41	87	1039
5.7	Permanent freshwater marshes and pools (<8 ha)	2001	1147	175	382	3705
5.8	Seasonal or intermittent freshwater marshes and pools (<8 ha)	1466	406	66	124	2062
5.9	Freshwater springs and oases	436	188	48	531	1203
5.10	Tundra wetlands (includes pools and temporary waters from snowmelt)	0	13	0	4	17
5.11	Alpine wetlands (includes temporary waters from snowmelt)	4	20	3	4	31
5.12	Geothermal wetlands	7	20	0	23	50
5.13	Permanent inland deltas	142	27	10	15	194
5.14	Permanent saline, brackish, or alkaline lakes	116	34	2	72	224
5.15	Seasonal or intermittent saline, brackish, or alkaline lakes and flats	40	17	1	16	74
5.16	Permanent saline, brackish, or alkaline marshes or pools	127	38	5	47	217
5.17	Seasonal or intermittent saline, brackish, or alkaline marshes and pools	60	23	1	12	96
5.18	Karst and other subterranean hydrological systems (inland)	130	4	311	335	780
	Total unique species	12,406	5176	2888	2406	22,876

*Note*: Some species are coded to multiple habitat classes.

### Species data

Species attributes (taxonomy, extinction risk, and habitat associations), point localities, and range maps were downloaded from the IUCN Red List 2022‐2 (IUCN, 2022). Data were downloaded for 12,995 extant fishes, 6078 odonates, 2612 decapod crustaceans (crayfishes, crabs, and shrimps), and 3424 mollusks that occurred in freshwater systems (freshwater [= inland waters] on the IUCN Red List) (25,109 species in total). These taxonomic groups were chosen because they rely on inland wetland habitats for all or part of their life cycle and are not accounted for in existing AOH mapping methodologies (e.g., mammals, birds, amphibians, and reptiles that are included in terrestrial AOH applications). Species were excluded if their habitat associations were unknown (1165 species), if their habitat associations described only nonwetland habitats (861 species), or if the species’ habitat associations were detailed only to the highest IUCN habitat class (level 1, e.g., IUCN habitat class 5 wetlands) rather than more specific habitat subclasses (level 2, e.g., 5.3 shrub‐dominated wetlands, 207 species). Data on the remaining 22,876 species (Table 1) were used to develop the methodology.

Range polygons were filtered based on presence (to include extant and probably extant) and origin (to include native, reintroduced, assisted colonization). Parts of the range where a species was possibly extinct were included if a species was critically endangered and possibly extinct (i.e., where possibly extinct polygons represented the entire species’ range). Species’ point localities were filtered based on presence (to include extant, probably extant, possibly extinct), origin (to include native, reintroduced, assisted colonization), and seasonality (to include resident, breeding, or nonbreeding).

Species point locality data from the IUCN Red List (318,984 points) were supplemented with data from the Global Biodiversity Information Facility (983,846 points) (GBIF, 2025) and the IUCN Species Survival Commission Dragonfly and Mollusc Specialist Groups (84,822 and 947 points, respectively) (V.C. and M. Seddon, personal , communication). For GBIF‐derived data, we selected only records for which coordinates were recorded to at least 3 decimal places (∼100 m coordinate precision), that were collected from 2012 to 2022, for which the species were recorded as present, and for which coordinate uncertainty was <100 m. The GBIF‐derived points were removed when the issues field in the GBIF metadata indicated potential inaccuracies in the point geometry, such as an invalid datum, presumed negated coordinates, or swapped coordinates.

Point locality data from all sources were assessed for potentially erroneous values and were removed if the coordinates fell outside the species’ mapped range, fell in seas or oceans, were exactly zero, had equal values for latitude and longitude, or coincided with country centroids, capital cities, or institutional addresses (implemented using CoordinateCleaner in R) (Zizka et al., 2019). We excluded species from model development that were reported to occur in more than 5 wetland habitats. We did this to represent both habitat generalists and specialists while reducing the noise introduced where species occurred in a large number of habitats. This reduced the risk of drawing false associations by chance and increased the likelihood of true associations reaching statistical significance. This resulted in 315,601 point locality observations from 2,397 species. From these, 80% were randomly selected and used to train the statistical model (i.e., the training set) and 20% were reserved for map validation.

### An empirical crosswalk to wetland‐cover classes

Twelve of the 18 inland wetland habitat classes from the IUCN Habitats Classification Scheme were translated into spatially explicit wetland‐cover classes following the method of Lumbierres, Dahal, Di Marco, et al. (2022). We used a beta version of the Global Lakes and Wetlands Dataset 2 (Lehner et al., 2025) in place of the Copernicus Global Land Service Land Cover (CGLS‐LC100) dataset. The IUCN habitat classes were shrub‐dominated wetlands (5.3), bogs, marshes, swamps, fens, and peatlands (5.4), permanent freshwater lakes over 8 ha (5.5), seasonal or intermittent freshwater marshes or pools under 8 ha (5.6), permanent freshwater marshes or pools under 8 ha (5.7), tundra wetlands (5.10), alpine wetlands (5.11), geothermal wetlands (5.12), permanent inland deltas (5.13), permanent saline, brackish, or alkaline lakes (5.14), seasonal or intermittent saline, brackish, or alkaline lakes and flats (5.15), and permanent saline, brackish, or alkaline marshes or pools (5.16).

The beta version of GLWD combines multiple data products to produce a global representation of 30 wetland ecosystem classes at approximately 500‐m resolution (Lehner et al., 2025). The GLWD includes rivers, lakes, and multiple other wetland types and incorporates information on seasonality, salinity, and whether they are natural or artificial, among other factors (Lehner et al., 2025). The GLWD represents global wetland cover as either the dominant class or the fractional cover per grid cell. We used the fractional cover of each wetland class. The final GLWD 2 has become available since the writing of this paper and has a slightly different configuration of classes (33 in total) (Lehner et al., 2025).

The fractional cover of each GLWD class surrounding each species point locality was modeled as a function of the presence or absence of each IUCN inland wetland habitat class associated with that species with logistic regression. Bilinear interpolation of the 4 nearest cells was used to assign a distance‐weighted average of fractional cover for each GLWD class at each species point locality. This was to account for any remaining uncertainty in point location and where points occurred close to the boundary between cells. The distance weighting meant that if a point locus coincided with a cell centroid exactly, the value attributed to the point was the value of the cell where the point fell. Three GLWD classes, saline lakes (518 points), arctic peatland (1008 points), and saltpans or brackish wetlands (1466 points), were excluded from the model due to low numbers of observations.

Separate logistic regression models were fitted, each with the fractional coverage of a different GLWD class as the response variable, via the lme4 package (Bates et al., 2015) in the statistical programming language R 4.2.1. The models were fitted using observations from all taxonomic groups and included 2 random effects: country in which the observation was recorded and species nested within taxonomic class. The final dataset used to run each model consisted of all training points where fractional coverage of the response GLWD class was >0, plus an equally sized random sample of points where the fractional cover of the response GLWD class was zero.

The strengths of associations were measured using odds ratios (ORs), calculated as the exponentiated coefficients from the logistic regression models. These represented the degree to which particular IUCN habitat classes coded for a species increase or decrease the likelihood that the species has point localities in a particular wetland‐cover class. Odds Ratios of <1 indicated a negative association, and values >1 indicated a positive association. Nonsignificant associations (i.e., where *p* > 0.05) were excluded. A threshold was used to convert the table of continuous ORs into a binary crosswalk table. Three candidate thresholds were chosen based on the tertiles of all significant positive associations (1.12, 1.66, 5.61). The thresholds reflect the minimum OR required for a particular GLWD wetland cover to represent habitat for species that occupy a particular IUCN habitat. The performance of each threshold was assessed, and the final threshold was chosen based on the results from map validation (Appendix S2–S4).

#### Thematic associations

Thematic associations were drawn between 4 IUCN inland wetland habitats and GLWD wetland‐cover classes to ensure that these associations were present regardless of the results of the empirical crosswalk. Permanent rivers and streams (IUCN class 5.1) and seasonal or intermittent rivers and streams (IUCN class 5.2) were jointly represented by GLWD classes rivers and streams (Lehner et al., 2025). Permanent freshwater lakes (IUCN class 5.5) and permanent saline, brackish, or alkaline lakes (IUCN class 5.14) were represented by GLWD classes freshwater lake and saline lake, respectively. The potential to further refine habitat for riverine species by river intermittence (Messager et al., 2021) and the potential to refine AOH by lake bathymetry were considered but not implemented due to data limitations (described in Appendix S1).

To represent habitat for caves and other subterranean hydrological systems (IUCN class 5.18), the World Karst Aquifer Map (WoKAM; Chen et al., 2017) was used. The suitability of WoKAM to represent this habitat class was investigated using 36 species with available point locality data of 506 species exclusively associated with caves and other subterranean habitats. Among all 36 karst‐exclusive species, 73% of points coincided with the WoKAM, including all available points for 26 species.

#### Unmapped habitats

Three habitat classes could not be reliably translated into any available spatial data on wetland cover and were therefore unmapped. Unmapped habitats were seasonal freshwater pools (IUCN class 5.8), springs and oases (IUCN class 5.9), and seasonal saline, brackish, or alkaline pools (IUCN class 5.17). The entire range for any species associated with an unmapped habitat was considered potential habitat (AOH) or was refined by elevation limits if available.

### AOH mapping process

For each species, the relevant wetland classes were identified via the IUCN Red List habitat coding and the full crosswalk table. Fractional AOH coverage was summed across all relevant land‐cover classes for each cell that intersected the species’ range up to a maximum of one. If no cells in the species’ range contained potential habitat, then all cells in the range were considered potential habitat, applying a precautionary approach to avoid species having no AOH map. Cells were further excluded if the cell elevation range did not overlap the species elevation range. AOH was refined by elevation for odonate species only, due to limited recording of elevation limits among other taxa.

For the validation based on point data to be effective, all wetland (IUCN class 5 inland wetlands) and nonwetland habitats for the species had to be included in AOH. Otherwise, it would be unclear whether points that fell outside the AOH were due to systematic errors in the method or because the species was observed in nonwetland habitats. Estuaries and reservoirs (IUCN classes 9.10 and 15.1) were represented by GLWD classes estuaries and reservoirs, respectively. Terrestrial habitats were mapped according to Lumbierres, Dahal, Di Marco, et al. (2022) (developed for mammals and birds), and marine habitats were mapped according to Turner et al. (2024) (developed for a range of taxa).

#### Preprocessing of spatial data inputs

All spatial input datasets were harmonized to a ∼1 × 1‐km raster grid (992.2927 × 992.2927 m specifically) in a Mollweide equal‐area global geographic projection. For all land‐ or sea‐cover datasets, this involved reprojecting the data at a resolution similar to the raw resolution (∼500 m for freshwater and terrestrial, ∼1 km for marine) with the nearest neighbor method and aggregating cell values to give the proportion of each land‐cover class per ∼1 × 1‐km cell. The raw terrestrial land‐cover data were structured as a single dominant class per cell, so it was reclassified before aggregation to give the proportion of each class per ∼1‐km cell. The WoKAM Karst aquifers were rasterized at ∼100‐m resolution before being aggregated to give proportional cell cover at ∼1‐km resolution. Species’ range polygons were rasterized at ∼1‐km resolution, whereby cells that intersected the range polygon were assigned a value of 1.

The FABDEM 1.2 (Hawker et al., 2022) was used to represent elevation. The FABDEM was re‐projected and aggregated to give 2 layers indicating the minimum and maximum elevation per ∼1‐km cell. Species’ elevation associations were modified to correct for coding errors, following Baisero (2021). Where either upper or lower elevation limit was missing or outside the range of global values present in the FABDEM data, the missing or incorrect limits were set to the global maximum (8580 m) or minimum (−427 m) FABDEM value, respectively. The same correction was applied where the lower elevation limit was higher than the upper elevation limit. Species’ elevation limits were also adjusted for species that had an elevation range less than the vertical accuracy of FABDEM. If the elevation range was <12 m, the upper and lower limits were widened by 6 m. These values were chosen based on the highest mean absolute error of FABDEM across different land‐cover classes (Hawker et al., 2022).

### AOH map validation

The AOH maps were validated following the 2‐stage method of Dahal et al. (2022). In Step 1, we assessed map accuracy based on map and point prevalence per species. Map prevalence is the proportion of the range represented by AOH, and point prevalence is the proportion of species’ point localities that fell where fractional coverage of AOH was >0. The fractional coverage of habitat surrounding each species point locality was determined using bilinear interpolation of the 4 nearest cells. Accuracy was calculated as the difference between point prevalence and map prevalence, where a positive accuracy (i.e., point prevalence greater than map prevalence) indicated a greater accuracy than expected if cells in the species’ range were allocated to AOH randomly. Point prevalence was calculated for the 20% of points withheld from the empirical crosswalk in addition to any points available for species coded to more than 5 inland wetland habitats (i.e., the validation set). Species were excluded from the validation set if they had fewer than 10 points available, resulting in a validation set of 219,045 points among 1395 species.

An additional 1557 fish species had IUCN Red List assessments with range maps published in late 2023 (IUCN, 2023); as such, they were not used to develop the empirical crosswalk. AOH maps were produced for these species based on the crosswalk developed on IUCN (2022), and the 998 additional species with at least 10 point localities were used to test AOH model performance on novel species (i.e., the testing set).

In Step 2, a logistic regression model predicting map prevalence was fitted to identify systematic errors in AOH map creation. The fitted predictors consisted of elevation range, elevation mid‐point, and number of habitats (at level 2 in the IUCN Habitats Classification Scheme). A random effect of family was used to account for unexplained taxonomic variation. The difference between the observed and expected map prevalence values was used to identify and investigate outliers. The Tukey's fence method of outlier detection was used; values were considered outliers if they were >1.5 times the interquartile range above the third or below the first quartile (Tukey, 1977). All 2114 AOH maps identified as outliers were investigated for potential systematic errors (Appendix S6). Any species suspected of having erroneous or missing habitat or elevation associations or incorrect range boundaries were reported to the IUCN Red List unit for consideration in future reassessments. The data and code to accompany this paper are available on request.

## RESULTS

### Association between IUCN inland wetland habitats and GLWD wetland cover

In total, 100 combinations of wetland cover and IUCN habitat showed a significant positive association (OR > 1 and *p* ≤ 0.05) and 39 showed a significant negative association (OR < 1 and *p* ≤ 0.05). The highest threshold was chosen because it showed the highest mean accuracy, with the lowest mean map prevalence and only a small reduction in point prevalence (Appendix S2).

At the highest and selected threshold of association, permanent freshwater lakes (IUCN class 5.5) were the only habitat class not associated with any GLWD classes (Appendix S5). Geothermal wetlands (IUCN class 5.12) and saline, brackish, or alkaline lakes (IUCN classes 5.14 and 5.15) were associated with the greatest numbers of GLWD classes (Appendix S5). Overall, 15 of the 27 included GLWD classes were not associated with any IUCN habitats at the selected threshold (Appendix S5). Three of the modeled IUCN inland wetland habitats each had over 1000 representative species. Permanent freshwater marshes or pools (IUCN class 5.7) were associated with ephemeral nonforested wetland; bogs, marshes, swamps, fens, and peatlands (class 5.4) were associated with paddy rice and palustrine regularly flooded nonforested; and seasonal freshwater lakes (IUCN class 5.6) were associated with paddy rice and ephemeral forested.

### AOH for freshwater species

AOH maps were generated for 19,867 species based on the full crosswalk table (Table 2). Of these, 2702 species occupied at least one unmapped habitat class, and for 221 species, no habitat in their range was identified. Of the remaining 16,944 species, the mean proportion of each species’ range that was AOH (i.e., map prevalence) was 18% (SD 13) among odonates, 22% (SD 21) among decapod crustaceans, 26% (SD 26) among fishes, and 32% (SD 30) among mollusks. A global map of the dominant IUCN inland wetland habitat per cell based on the crosswalk was also generated (Appendix S17).

**TABLE 2:** The full crosswalk table relating International Union for Conservation of Nature (IUCN) Red List inland wetland habitats to wetland cover classes.

Habitat class (code)	Wetland cover class^a^
Permanent rivers, streams and creeks (5.1)	4, river; 7, small streams
Seasonal rivers, streams and creeks (5.2)	4, river; 7, small streams
Shrub‐dominated wetlands (5.3)	20, ephemeral forested; 30, paddy rice
Bogs, marshes, swamps, fens, peatlands (5.4)	17, palustrine regularly flooded nonforested; 30, paddy rice
Permanent freshwater lakes (5.5)	1, freshwater lake
Seasonal freshwater Lakes (5.6)	20, ephemeral forested; 30, paddy rice
Permanent freshwater pools (5.7)	21, ephemeral nonforested
Seasonal freshwater marshes or pools (5.8)	Not mapped
Freshwater springs and oases (5.9)	Not mapped
Tundra wetlands (5.10)	16, palustrine regularly flooded forested; 20, ephemeral forested; 30, paddy rice
Alpine wetlands (5.11)	7, small streams; 17, palustrine regularly flooded non forested; 21, ephemeral nonforested
Geothermal wetlands (5.12)	7, small streams; 8, lacustrine forested; 9, lacustrine non forested; 21, ephemeral nonforested; 30, paddy rice
Permanent inland deltas (5.13)	17, palustrine regularly flooded nonforested; 19, palustrine seasonally saturated nonforested; 20, ephemeral forested; 30, paddy rice
Permanent saline, brackish, or alkaline lakes (5.14)	2, saline lake; 16, palustrine regularly flooded forested; 17, palustrine regularly flooded nonforested; 20, ephemeral forested; 21, ephemeral nonforested; 30, paddy rice
Seasonal saline, brackish, or alkaline lakes (5.15)	7, small streams; 13, riverine seasonally flooded non forested; 16, palustrine regularly flooded forested; 21, ephemeral non forested; 23, temperate peatland; 30, paddy rice
Permanent, saline, brackish, or alkaline pools (5.16)	20, ephemeral forested
Seasonal saline, brackish, or alkaline pools (5.17)	Not mapped
Karst and other subterranean hydrological systems (5.18)	31, karst
Estuaries (9.1)	5, estuarine river
Water storage areas >8 ha (15.1)	3, reservoir

^a^ Wetland cover classes 1–30 are from the Global Lakes and Database (beta version 2), and class 31 is from the World Karst Aquifer Map.

**FIGURE 2:**
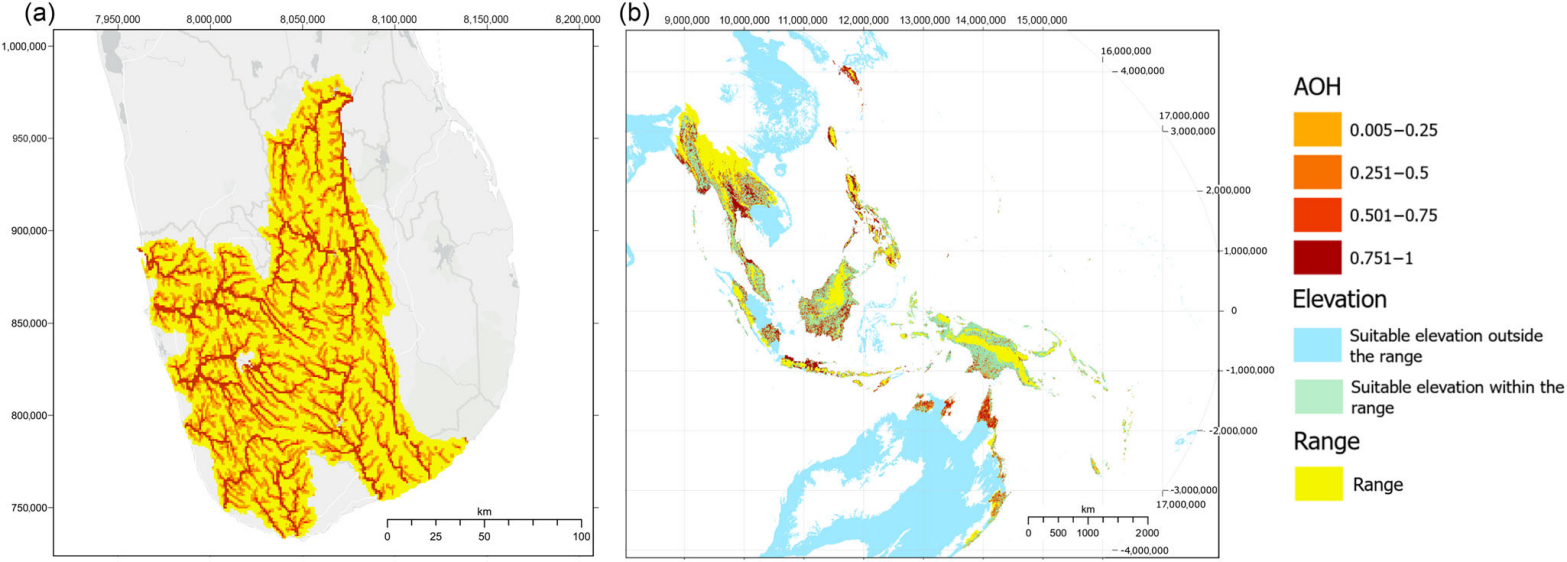
The fractional coverage of area of habitat (AOH) in the range of (a) *Belontia signata* (Günther, 1861) in Sri Lanka and (b) *Agrionoptera insignis* (Rambur, 1842) across Asia Pacific. The AOH is the proportion of each ∼1 × 1 km cell covered by habitat and presented in Mollweide equal‐area global geographic projection. A basemap (Esri, 2017) was used in (a) to distinguish between land and sea areas.

Example maps for 2 species are provided to demonstrate how AOH maps were generated. *Belontia signata* (Günther, 1861), a fish that exclusively occupies permanent rivers, streams, and creeks (IUCN class 5.1), had a map prevalence of 17% and a point prevalence of 82% (Figure 2a). *Agrionoptera insignis* (Rambur, 1842), an odonate associated with bogs, marshes, swamps, fens, and peatlands (class 5.4) and permanent freshwater marshes or pools (class 5.7) and with an elevation range of 350 m (0–350 m), had a map prevalence of 18% and a point prevalence of 82% (Figure 2b).

### Validation of AOH maps

A total of 1395 species had sufficient point locality data for AOH validation based on point prevalence (10% of all maps generated). This represented 6.5% of fishes, 13.9% of odonates, 1.2% of decapod crustaceans, and 3.6% of mollusks. Mean point prevalence was 0.83 (SD 0.22) among odonates, 0.73 (0.24) among decapod crustaceans, 0.92 (SD 0.12) among fishes, and 0.87 (SD 0.16) among mollusks.

**FIGURE 3:**
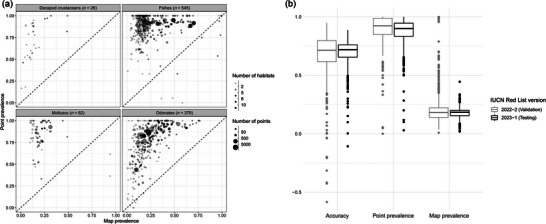
Validation of area of habitat (AOH) maps: (a) relationship between point prevalence and map prevalence among taxonomic groups for all 1003 species that could be validated using point data and data from International Union for Conservation of Nature (IUCN) Red List 2022‐2 that are not associated with any unmapped habitat classes and (b) relative performance of the AOH mapping method compared with fish species used to train and validate the model (IUCN Red List 2022‐2) and additional fish species newly assessed in IUCN Red List 2023‐1 (map prevalence, proportion of a species’ range that is AOH; point prevalence, proportion of species’ point localities in the AOH; accuracy, difference between point prevalence and map prevalence; positive values [point prevalence higher than map prevalence], maps with a higher accuracy than expected if AOH were distributed randomly; boxes, median and interquartile range [IQR]; whiskers, 1.5 times IQR).

Based on the difference between map prevalence and point prevalence per species (i.e., map accuracy), AOH maps had above‐random accuracy for 1095 out of 1395 species (78%) (Figure 3a) and an overall mean accuracy of 0.44 (SD 0.33). The performance increased when excluding maps for the 392 species that occupied at least one unmapped habitat class. AOH maps for 987 of the remaining 1003 species (98%) had above‐random accuracy (100% of decapod crustaceans, 99% of fishes, 97% of mollusks, and 98% of odonates). Mean accuracy was 0.61 (SD 0.21) overall and 0.55 (SD 0.19) for decapod crustaceans, 0.67 (SD 0.2) for fishes, 0.62 (SD 0.26) for mollusks, and 0.53 (SD 0.18) for odonates. All species with sufficient point locality data for validation had some habitat in their range.

Of the 1557 additional fish species that were recently assessed in IUCN Red List 2023‐1, 80 species occupied at least one unmapped habitat and 53 had no habitat identified in their range. For the remaining 1424 species, 686 had at least 10 point localities and were used to test model performance. Mean point prevalence was 0.87% (SD 0.12), mean accuracy was 0.70 (SD 0.11), and all species had above‐random accuracy. Therefore, the AOH mapping method performed similarly well on newly assessed species compared with species used to train the model (Figure 3b).

## DISCUSSION

We demonstrated a method to translate IUCN inland wetland habitat classes into geographic wetland cover, enabling the mapping of AOH for decapod crustaceans, fishes, odonates, and mollusks. With a mean map prevalence of just 18–32%, 73–92% of species’ point localities were on these species’ AOH maps. Furthermore, the method performed equally well when tested against species for which no data were included in method development. These results suggest that our method is useful for producing a more refined representation of the distribution of species occupying inland wetland habitats.

Our AOH mapping method performed similarly well to existing AOH mapping methods developed for terrestrial taxa. For terrestrial vertebrates, AOH comprised 55–79% of the area of mapped ranges (map prevalence), with 77–94% of observation points in the predicted AOH (point prevalence) and 79–95% of maps having greater‐than‐random accuracy (Dahal et al., 2022; Ficetola et al., 2015; Lumbierres, Dahal, Soria, et al., 2022; Rondinini et al., 2011). Our models also predicted that AOH comprised a lower proportion of the area of mapped ranges, which was expected given that inland wetlands cover a relatively small proportion of Earth's surface (Lehner & Döll, 2004) and that ranges are mapped to catchments that can include large areas where there is no habitat. However, such mapping to catchments may better represent inferred and projected occurrences of the species that range maps, by definition, should include (IUCN, 2021).

With our AOH mapping method, we sought to minimize the exclusion of areas where the species could be present (omission error); therefore, several methodological decisions were taken that may overestimate the AOH (commission error). Each species’ mapped range was rasterized at ∼1‐km resolution, where any cell intersecting the species’ range polygon was included. This approach was chosen to accommodate spatial inaccuracies, avoid excluding potential habitat around the edges of species’ ranges, and increase the likelihood that some AOH was mapped for species with extremely small ranges. This means there may be areas mapped as AOH that would fall outside the range limits if the range were rasterized based on cell centroids or at a finer resolution. In modeling the association between habitats and land cover, we did not correct *p* values for multiple testing (e.g., with Bonferroni correction) because each model tested a distinct hypothesis on a distinct dataset. However, because the datasets were not entirely independent, the use of such a correction is defensible, noting that such techniques can increase the risk of type 2 errors (Cabin & Mitchell, 2000). Furthermore, the minimum and maximum elevation per cell were used to assess whether the cell contained suitable elevation for the species. Although this removed the chance that AOH was falsely excluded in areas of highly variable elevation, it represents another potential source of overestimation. Other AOH mapping applications opted to use a single value for elevation per cell but also produced maps at a higher output resolution (Lumbierres, Dahal, Soria, et al., 2022), so the within‐cell variability in elevation was lower.

Understanding the commission error associated with these methodological choices requires data on species’ absence, which are not widely available for the taxa studied. Efforts have been made to predict species’ pseudoabsence by accounting for survey effort (Johnston et al., 2021). Applying such methods in the validation of AOH maps presents a potential avenue for the continued improvement of AOH mapping methods in freshwater and other realms.

For 1.1% of species, no habitat was identified in the species’ range based on the highest threshold of association between IUCN habitat and GLWD wetland‐cover classes. Dahal et al. (2022) similarly failed to find relevant land cover for some terrestrial bird and mammal species with very small ranges. These methods allow a different threshold of association to be chosen as per the needs of the user and application. For example, a lower minimum threshold might be chosen if the priority is to map AOH across a group of small‐ranged species or for fewer taxonomic groups. However, the aim here was to develop a methodology that was reproducible across a wide diversity of taxa, so the threshold with the highest overall accuracy across all included groups was chosen. Species for which no available AOH was found could also indicate errors in the habitats and elevation or range limits reported by IUCN Red List assessors.

The large differences in model performance between species occupying unmapped habitat and those for which IUCN inland wetland habitats could be translated into GLWD wetland cover reinforce the importance of high‐quality datasets of the distribution of inland wetlands. In particular, seasonal pools (IUCN classes 5.8 and 5.17), which include waterbodies up to 8 ha, could not be mapped. Accurately predicting the spatial distribution of these habitats is challenging due to their temporal and spatial heterogeneity and association with a large variety of surrounding land‐cover classes (Varin et al., 2021). However, early attempts to identify the environmental correlates (Campbell Grant, 2005) and predict their occurrence either statistically (Cormier et al., 2013) or with LiDAR data (Varin et al., 2021) have shown encouraging results. Therefore, technical advances may make it possible to include these habitats in AOH mapping methods in the future. Further distinction between ephemeral or vernal pools (<1 ha) (Calhoun et al., 2005) and other small seasonal waterbodies (1–8 ha) in future revisions of the IUCN habitat classification scheme may further enable AOH mapping for these species. A similar challenge is that some species use small water‐filled cavities in terrestrial habitats, such as in plants (phytotelmata) (Orr, 1994), but currently there are no methods to map these. By developing strategies to integrate AOH mapping across all inland areas, solutions to predicting such small‐scale freshwater habitats may be found.

Strong associations were found between rice paddies and multiple IUCN inland wetland habitat classes. The increased survey effort and detectability of species in human‐modified landscapes likely contributed to the strong association observed (Lumbierres, Dahal, Di Marco, et al., 2022; Meyer et al., 2015). Nevertheless, the extent of artificial wetlands, predominantly rice paddies, almost doubled between 1970 and 2015 and is now estimated to represent 6.7–12% of wetland cover (Gardner & Finlayson, 2018; Lehner et al., 2025). Paddy fields support a moderate to high level of biodiversity (Gardner & Finlayson, 2018) that relies on the suitability of, and connectivity with, the surrounding environment. This emphasizes the critical role that artificial habitats can play in the conservation of some species, especially given that human land‐use change and demands on food production continue to increase (IPBES, 2019; Venter et al., 2016). However, with changes in cultivation practices and land use in the surrounding landscapes, the value of rice paddies as habitat for species is declining in some regions (Luo et al., 2014).

The increased thematic resolution of this method for mapping inland wetland habitats will have great benefits for the representation of aquatic species in conservation policy and practice. AOH maps are used in a variety of processes, including estimates of species richness or population size (Rompré et al., 2009; Sutton et al., 2023), assessments of extinction risk (Brooks et al., 2019), identification of key biodiversity areas (IUCN, 2016) or other important sites for biodiversity (Dória & Dobrovolski, 2021), and determination of metrics to support action under and measurement of progress toward science‐based targets (e.g., the STAR metric [Mair et al., 2021; Turner et al., 2024]). Therefore, this method not only allows more accurate mapping of AOH for freshwater fauna but also facilitates their inclusion in conservation processes in which they have been historically underrepresented.

Ours is the first attempt to map AOH in a way that is tailored for inland wetland habitats. It considerably reduced the area that was predicted to be suitable for each species relative to range maps and successfully predicted the presence of habitat for most points where species had been documented to occur. Although the method was developed using data for freshwater fishes, decapod crustaceans, odonates, and mollusks, we believe it can be applied to other inland wetland‐associated species. Testing its performance on such species would be a beneficial next step. Development of our method was made possible by the recent availability of high‐quality datasets on the distribution of relevant wetland cover. However, gaps remain for mapping small seasonal waterbodies, springs, oases, and some artificial habitats. Methods for predicting the occurrence of such features could enable the accurate refinement of AOH predictions for even more species. Work is now underway to operationalize the integration of methods for mapping freshwater, terrestrial, and marine AOH and the production of more comprehensive AOH datasets for a wider range of species. These will enable more accurate biodiversity analyses and more effective conservation planning.

## AUTHOR CONTRIBUTIONS

All authors contributed to conceptualization either through the 2 scoping workshops or through the ongoing work to finalize the methodology and manuscript. Thomas M. Brooks and Nicholas B. W. Macfarlane contributed to funding acquisition. Bernhard Lehner, Günther Grill and Viola Clausnitzer contributed to data curation. Thomas Starnes, Catherine A. Sayer, Daniele Baisero, Bernhard Lehner, Thomas A. Worthington, Stuart H. M. Butchart, Arnout van Soesbergen, Louise Mair, and Francesca A. Ridley contributed to the design of the analysis and validation that was performed by Francesca A. Ridley. Visualizations and the initial draft manuscript were produced by Francesca A. Ridley. All authors contributed to reviewing and editing the final manuscript. Thomas Starnes supervised the project.

## Supporting information



Supplementary Materials.

Supplementary Materials.

Supplementary Materials.
