# Supplementary material for: Mapping area of habitat for inland wetland species
Source: Conserv Biol. 2025 Oct 29;40(1):e70163. doi: 10.1111/cobi.70163 (PMC12856809; doi:10.1111/cobi.70163)
Supplement: Supplementary file 1 — Supplementary Materials. [file COBI-40-e70163-s002.docx]

Supplementary information on translating IUCN Inland Wetland Habitats into spatially explicit wetland cover

## Appendix S1. Mappable habitat characteristics that were explored but not included in the AOH mapping methodology

### Seasonality of rivers and streams

The potential to further refine suitable habitat for riverine species by incorporating river attributes, such as seasonal intermittence and flow rate (Messager *et al.* 2021), was explored but not implemented because species coded as exclusively occupying permanent rivers on the IUCN Red List were frequently observed in rivers of the highest likelihood to cease flowing for 30 days of the year according to the Messager *et al.* (2021) data. Furthermore, relatively few species were coded exclusively to seasonal rivers (52 of 2,499 seasonal riverine species) and among these there was insufficient availability of point presence data (113 points among 7 species) to investigate whether their observed presence was explained by river flow/intermittence attributes.

### Lake bathymetry

The refinement of AOH by lake bathymetry for permanent lake species was considered but not applied due to the disproportionately low number of species for which no depth limits were available (4,574 of 4,790 species associated with IUCN habitat 5.5: Permanent Freshwater Lakes) and the amount of work required to assemble a global lake bathymetry dataset, which was deemed outside the scope. Further uncertainty over how to apply depth associations is introduced where species occur in both marine and lake habitats. Some 279 named lakes were identified within the ranges of the 156 lacustrine (lake-dwelling) species that had depth limits available and did not also occur in marine habitats. Bathymetry data for the largest of these lakes were sparsely available and where available were presented in a combination of different data types and formats Leon & Cohen (2012). For some species the mapped range was already refined by bathymetric limits and so, for these species, depth was accounted for.

## Appendix S2. Threshold selection for the empirical crosswalk between IUCN inland wetland habitats and GLWD v2

### Background and methods

A logistic regression model was used to empirically translate IUCN inland wetland habitats into GLWD v2 wetland cover classes (Lehner, B. unpublished data), from which the exponentiated coefficients (odds ratios, Table 1) were used to quantify the strengths of associations. A threshold was used to convert the quantitative associations into a binary table (crosswalk) indicating which GLWD v2 classes were used to represent each IUCN inland wetland habitat. This threshold indicated the minimum odds ratio required for a particular GLWD v2 class to represent a particular habitat.

Three candidate thresholds were chosen based on the tertiles of all significant positive associations from the logistic regression model (Odds ratios: 1.12, 1.66, and 5.61). The highest threshold represented the highest odds ratio required for a particular wetland cover to be mapped as suitable habitat for species that occupy a particular IUCN habitat. AOH maps were produced using each of the three candidate thresholds and the performance of each threshold was assessed by comparing the results from map validation using map prevalence and point prevalence.

Map prevalence was measured as the proportion of range cells that contained AOH, and point prevalence was the proportion of species’ point localities that fell within cells where AOH was present. The proportional coverage of AOH in each cell was extracted to species point localities using bilinear interpolation, a weighted average of the four nearest cells. If the average coverage of AOH was greater than zero that point was considered to be within the AOH. AOH maps where point prevalence was greater than map prevalence indicated a greater accuracy than expected if cells within the species’ range were allocated to AOH randomly.

AOH maps for the 997 species that were not associated with any ‘unmapped’ habitats (Supplementary File 2) and had at least 10 observation points were used to compare between the three candidate thresholds.

### Results of comparison among thresholds

In total, 100 combinations of wetland cover and IUCN habitat showed a significant positive association (odds ratio > 1 and p $\leq$ 0.05) and 39 showed a significant negative association (odds ratio < 1 and p $\leq$ 0.05). Odds ratios (OR) for significantly positive associations ranged from 1.12 to 723,905. Of the three candidate thresholds, the highest (Threshold 3, OR $\geq$ 5.61) included 34 coefficients, the middle threshold (Threshold 2, OR $\geq$ 1.66) included 67 coefficients, and the lowest (Threshold 1, OR $\geq$ 1.12) included 100 coefficients.

The highest threshold (Threshold 3) had the highest mean accuracy, with the lowest mean map prevalence and only a small decrease in mean point prevalence, relative to the lower thresholds (Table 2, Fig 1). Therefore, threshold 3 was chosen as the final accepted threshold to compute the binary crosswalk table.

# References

Hamilton, S. (2021). GLWNB Soundings and Bathymetry. Retrieved May 26, 2023, from <https://dataverse.harvard.edu/citation?persistentId=doi:10.7910/DVN/ITCOGT>

Leon, J.X. & Cohen, T.J. (2012). Improved digital elevation model (DEM) for Lake Eyre, Australia. Retrieved May 26, 2023, from <https://doi.pangaea.de/10.1594/PANGAEA.783838>

Messager, M.L., Lehner, B., Cockburn, C., Lamouroux, N., Pella, H., Snelder, T., Tockner, K., Trautmann, T., Watt, C. & Datry, T. (2021). Global prevalence of non-perennial rivers and streams. *Nature*, **594**, 391–397.

*Appendix S3. Summary of three candidate thresholds used to associate IUCN Inland Wetland Habitat classes with Global Lakes and Wetlands cover classes. Accuracy was measured as the difference between point prevalence and map prevalence where values above zero indicate above random accuracy and 1 represents maximum accuracy.*

|  | Threshold 1 | Threshold 2 | Threshold 3 |
| --- | --- | --- | --- |
| Mean point prevalence (SD) | 0.90 (0.15) | 0.87 (0.16) | 0.84 (0.19) |
| Mean map prevalence (SD) | 0.34 (0.22) | 0.29 (0.19) | 0.20 (0.15) |
| Mean accuracy (SD) | 0.56 (0.21) | 0.58 (0.21) | 0.63 (0.2) |


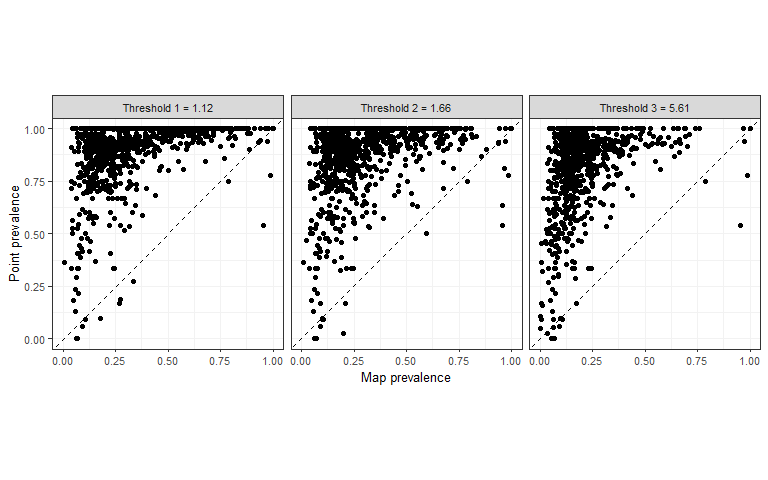
 *Appendix S4. The relationship between point prevalence and map prevalence among thresholds. Maps where point prevalence is greater than map prevalence are considered to be more accurate than expected if AOH were allocated among cells at random.*

Appendix S5. Odds ratios (OR) from the logistic regression models indicating the association between IUCN habitat coding and geographic wetland cover classes. Light blue = Threshold 1 (OR $\geq$ 1.12), medium blue = Threshold 2 (OR $\geq$ 1.66), and dark blue = Threshold 3 (OR $\geq$ 5.61).

|  |  | IUCN Habitat Class | | | | | | | | | | | |
| --- | --- | --- | --- | --- | --- | --- | --- | --- | --- | --- | --- | --- | --- |
| Wetland cover  class (GLWD v2) | Points | Shrub dominated wetlands (5.3) | Bogs, marshes, swamps, fens, peatlands (5.4) | Permanent freshwater lakes  (5.5) | Seasonal Freshwater Lakes  (5.6) | Permanent Freshwater Pools (5.7) | Tundra wetlands (5.10) | Alpine wetlands (5.11) | Geothermal Wetlands (5.12) | Permanent inland deltas (5.13) | Permanent Saline, brackish, or alkaline lakes (5.14) | Seasonal Saline, brackish, or alkaline lakes (5.15) | Permanent, Saline, Brackish or alkaline pools (5.16) |
| 7: Small streams | 121,382 |  | 0.42 | 4.72 | 0.54 | 0.47 |  | 30.14 | 97.88 | 0.03 | 0.65 | 14.01 | 1.90 |
| 14: Riverine seasonally saturated forested | 89,327 | 1.27 | 1.26 | 0.95 | 1.12 |  | 1.56 | 0.40 | 0.44 | 1.45 |  |  | 0.27 |
| 15: Riverine seasonally saturated non forested | 85,411 |  | 1.19 | 1.33 | 1.25 | 1.19 |  |  |  |  | 1.66 |  |  |
| 23: Temperate peatland | 74,043 | 0.60 | 5.38 |  | 0.54 | 1.52 | 1.58 | 0.59 |  | 3.92 |  | 23.48 | 0.02 |
| 12: Riverine seasonally flooded forested | 68,747 |  | 1.23 | 0.51 | 1.72 | 0.81 |  | 0.14 |  | 1.75 | 1.74 |  | 0.46 |
| 13: Riverine seasonally flooded non forested | 54,829 |  | 1.63 | 1.46 | 1.64 | 0.87 | 0.04 |  |  |  |  | 16.10 | 0.54 |
| 10: Riverine regularly flooded forested | 54,141 | 1.42 |  |  |  | 1.27 |  |  | 2.37 |  |  |  | 0.53 |
| 6: Other permanent waterbody | 53,916 |  | 0.79 | 0.69 |  |  |  |  |  | 4.68 | 1.90 | 3.98 | 2.45 |
| 11: Riverine regularly flooded non forested | 50,238 | 1.48 | 0.63 |  |  | 1.32 |  |  | 4.43 | 0.28 |  |  |  |
| 4: River | 38,608 |  | 0.67 |  |  |  |  | 4.88 |  |  |  |  |  |
| 8: Lacustrine forested | 37,748 | 0.54 | 1.49 | 1.70 | 2.14 | 0.59 | 1.60 |  | 5.92 | 0.06 |  |  | 0.38 |
| 9: Lacustrine non forested | 37,478 | 1.96 | 0.68 |  |  |  |  | 3.65 | 5.61 |  | 1.93 |  |  |
| 24: Tropical peatland | 37,266 |  | 1.55 | 0.68 |  |  |  |  |  |  |  |  |  |
| 28: Other coastal wetland | 36,690 | 1.26 | 1.17 |  | 1.66 | 1.17 |  |  |  |  | 1.39 | 2.90 | 1.50 |
| 18: Palustrine seasonally saturated forested | 32,904 |  |  | 0.69 |  | 1.34 |  |  |  |  |  |  |  |
| 1: Freshwater lake | 29,363 |  |  | 3.66 | 1.35 | 0.75 |  |  |  |  |  |  |  |
| 21: Ephemeral non forested | 26,734 | 3.83 |  |  |  | 76.04 |  | 9.17 | 11.36 |  | 15.17 | 164.26 |  |
| 19: Palustrine seasonally saturated non forested | 24,212 |  |  |  | 3.22 | 2.45 |  |  |  | 6.12 |  |  | 2.68 |
| 20: Ephemeral forested | 23,800 | 17.80 |  |  | 6.32 | 0.19 | 71.32 |  |  | 31.71 | 8.45 |  | 14.07 |
| 16: Palustrine regularly flooded forested | 22,108 |  |  |  | 2.82 |  | 7.11 |  |  |  | 6.10 | 21.05 |  |
| 17: Palustrine regularly flooded non forested | 17,614 |  | 7.31 |  |  |  |  | 175.07 |  | 16.15 | 31.00 |  |  |
| 3: Reservoir | 9,441 |  |  | 1.45 |  |  |  |  |  |  |  |  | 0.54 |
| 30: Paddy rice | 6,793 | 63.23 | 651.89 |  | 50.24 | 0.24 | 26.79 |  | 1,190.47 | 7.61 | 116.66 | 723,905.22 | 0.00 |
| 26: Saltmarsh | 6,735 |  | 1.91 |  | 3.40 |  |  |  |  |  |  |  |  |
| 25: Mangrove | 3,647 |  |  |  |  |  |  |  |  |  | 2.84 | 2.88 |  |
| 5: Estuarine river | 3,255 |  | 0.62 |  |  |  |  |  |  | 4.53 |  | 2.48 |  |
| 27: Delta | 2,949 |  |  |  | 1.41 | 1.58 |  |  |  |  |  |  |  |
