# Supplementary material for: Mapping area of habitat for inland wetland species
Source: Conserv Biol. 2025 Oct 29;40(1):e70163. doi: 10.1111/cobi.70163 (PMC12856809; doi:10.1111/cobi.70163)
Supplement: Supplementary file 2 — Supplementary Materials. [file COBI-40-e70163-s003.docx]

Outliers and species with no suitable habitat

# Appendix S6. Outliers in predicted map prevalence

In step two of model validation, a logistic regression model predicting map prevalence was fitted to identify systematic errors in AOH map creation. The fitted predictors consisted of elevation range, elevation mid-point, and the number of habitats (at level 2 in the IUCN Habitats Classification Scheme). A random effect of family was used to account for unexplained taxonomic variation. The difference between the observed and expected map prevalence values was used to identify and investigate outliers. The Tukey’s fence method of outlier detection was used, where values were considered outliers if they were more than 1.5 times the interquartile range above the 3rd or below the 1st quartiles.

The logistic regression model in total explained 23.4% of the variation in map prevalence and 23.2% was explained by the fixed predictors alone. Applying Tukey’s fence method to the difference between the observed and predicted map prevalence identified 2,114 species as outliers out of 16,944 species’ AOH maps for which no special adjustments were applied. Special adjustments were applied where species occupied at least one un-mapped habitat or where no cells with suitable wetland cover within the range were identified, as described in sections 3.3.4 and 3.4.2 of the main text. Assessing the distribution of outlier species among taxonomic groups, habitats, extinction risk categories, elevation ranges and elevation limits revealed that 47% of karst species (IUCN habitat class 5.18) and 42% of permanent freshwater lake species were outliers (Fig 1).


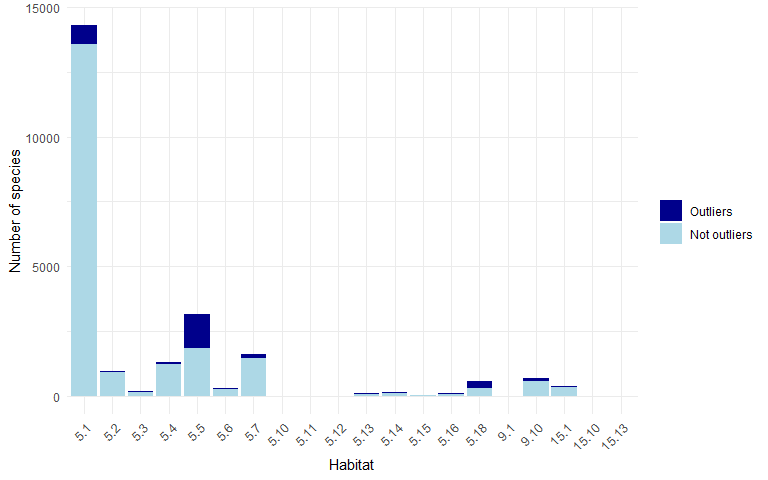
 Appendix S7. The distribution of species among freshwater (Inland Wetland) habitats


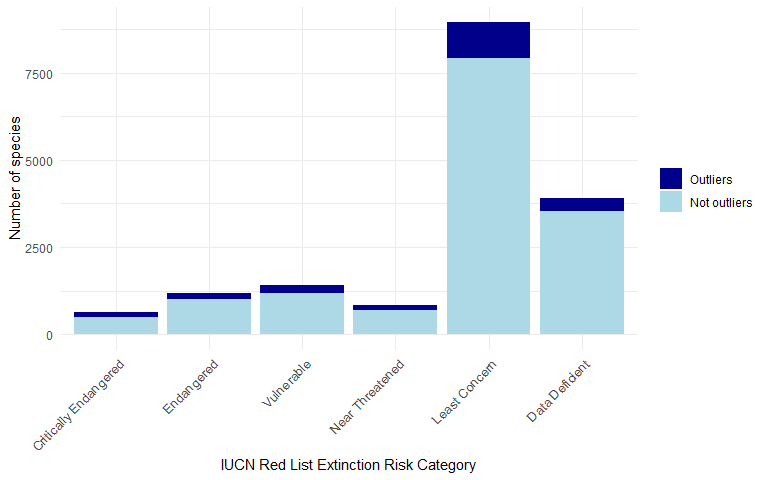


Appendix S8. The distribution of species among red list categories

Appendix S9. Proportion of outlier species per IUCN inland wetland habitat class

| Code | Habitat | Proportion of species   that were outliers |
| --- | --- | --- |
| 5.18 | Wetlands (inland) - Karst and Other Subterranean Hydrological Systems (inland) | 0.47 |
| 5.5 | Wetlands (inland) - Permanent Freshwater Lakes (over 8ha) | 0.42 |
| 5.13 | Wetlands (inland) - Permanent Inland Deltas | 0.34 |
| 5.15 | Wetlands (inland) - Seasonal/Intermittent Saline, Brackish or Alkaline Lakes and Flats | 0.33 |
| 5.14 | Wetlands (inland) - Permanent Saline, Brackish or Alkaline Lakes | 0.29 |
| 15.13 | Artificial/Marine - Mari/Brackishculture Ponds | 0.29 |
| 5.6 | Wetlands (inland) - Seasonal/Intermittent Freshwater Lakes (over 8ha) | 0.16 |
| 9.10 | Marine Neritic - Estuaries | 0.15 |
| 5.11 | Wetlands (inland) - Alpine Wetlands (includes temporary waters from snowmelt) | 0.14 |
| 5.16 | Wetlands (inland) - Permanent Saline, Brackish or Alkaline Marshes/Pools | 0.13 |
| 5.10 | Wetlands (inland) - Tundra Wetlands (incl. pools and temporary waters from snowmelt) | 0.11 |
| 5.3 | Wetlands (inland) - Shrub Dominated Wetlands | 0.09 |
| 5.7 | Wetlands (inland) - Permanent Freshwater Marshes/Pools (under 8ha) | 0.09 |
| 15.1 | Artificial/Aquatic - Water Storage Areas (over 8ha) | 0.08 |
| 5.12 | Wetlands (inland) - Geothermal Wetlands | 0.08 |
| 5.4 | Wetlands (inland) - Bogs, Marshes, Swamps, Fens, Peatlands | 0.07 |
| 5.2 | Wetlands (inland) - Seasonal/Intermittent/Irregular Rivers/Streams/Creeks | 0.06 |
| 5.1 | Wetlands (inland) - Permanent Rivers/Streams/Creeks (includes waterfalls) | 0.05 |


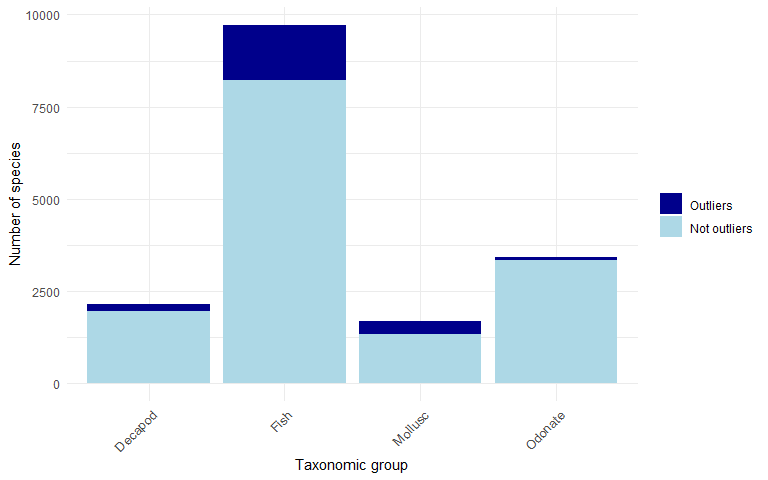


Appendix S10. The distribution of species among taxonomic groups


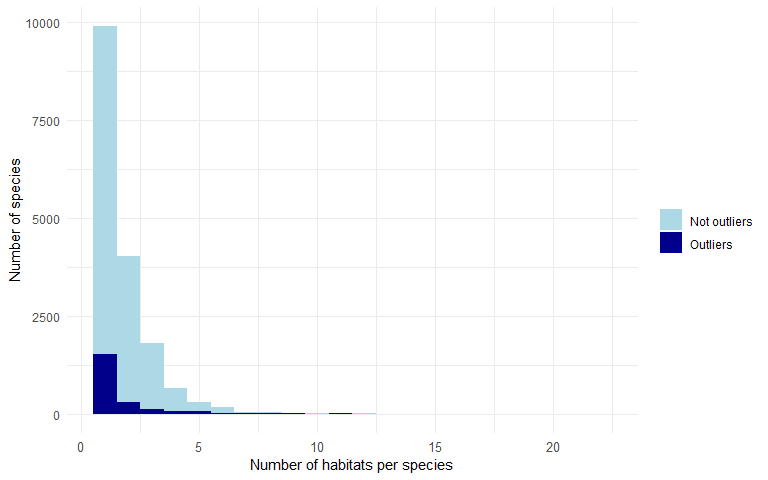


Appendix S11. Numbers of habitats per species compared between species found to be outliers and those that weren’t


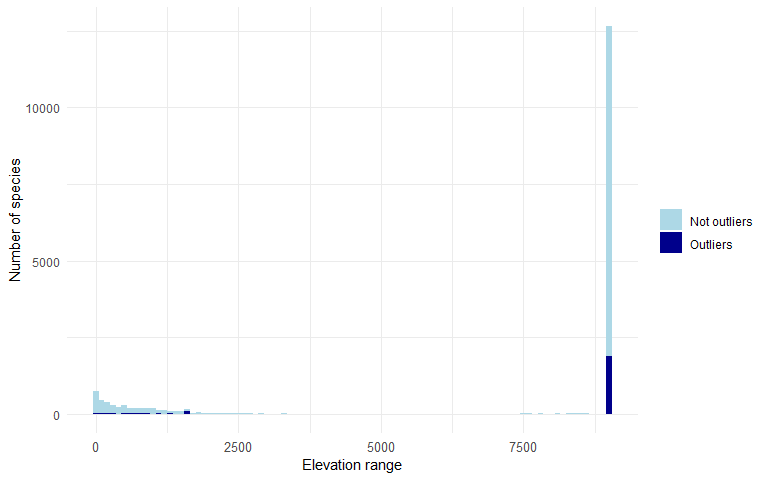


Appendix S12. Elevation range of species (in 100m intervals) compared between outlier and non-outlier species. Species with missing data or errors in their elevation preferences were replaced with the full elevation range of the Forest And Buildings adjusted Digital Elevation Model (FABDEM).


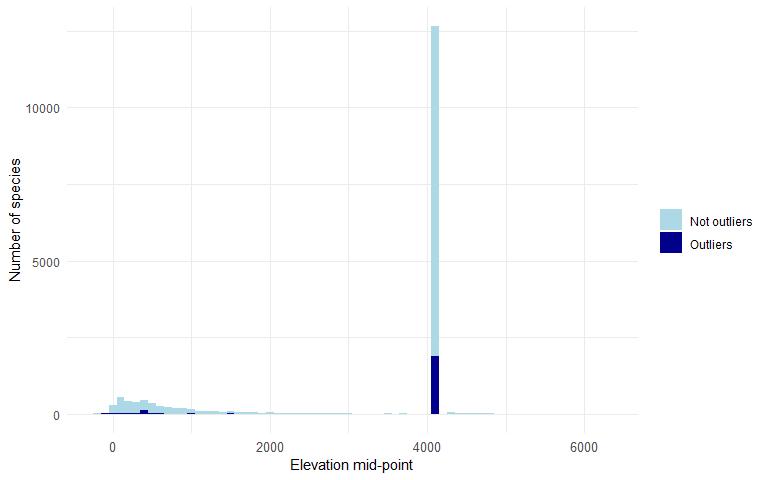


Appendix S13. Elevation mid-point of species (in 100m intervals) compared between outlier and non-outlier species. Species with missing data or errors in their elevation preferences were replaced with the full elevation range of the Forest And Buildings adjusted Digital Elevation Model (FABDEM).

# Appendix S14. Species for which no suitable habitat was identified within the range

For 211 species, no suitable habitat within the range was identified. For 10 species this was due to having no Extant or Possibly Extant range polygons, only Possibly extinct or Possibly Extant range polygons. Three of these species were Data Deficient: (*Procambarus connus*, *Nitia chefneuxi*,*Coeliccia resecta*), four were Endangered (*Allodontichthys hubbsi*, *Atherinella venezuelae*, *Drepanosticta marsyas* and *Rhinocypha latimacula*), two were Vulnerable (*Tiaroga cobitis* and *Macromia mnemosyne*) and one was Least Concern (*Auriglobus amabilis*). *Atherinella venezuelae* had Extant points but no Extant range.

Some species for which suitable habitat was identified had range sizes as small, sometimes smaller, than those for which no suitable habitat was identified (Table 2). However, the average range size among species for which no suitable habitat was identified was smaller than for species which suitable habitat was identified (Table 2).

Appendix S15. Relative range sizes for species with refined AOH maps vs species for which no AOH was identified and reverted to range

|  | Range size (Number of ~1km cells) | | | | |
| --- | --- | --- | --- | --- | --- |
| notes | median | mean | min | max | n |
| AOH reverted to range | 1,140.00 | 4,229.21 | 2 | 165,614 | 211 |
| AOHmapped | 18,217.00 | 415,151.08 | 1 | 39,343,202 | 16,944 |
