# Supplementary material for: Mapping area of habitat for inland wetland species
Source: Conserv Biol. 2025 Oct 29;40(1):e70163. doi: 10.1111/cobi.70163 (PMC12856809; doi:10.1111/cobi.70163)
Supplement: Supplementary file 3 — Supplementary Materials. [file COBI-40-e70163-s001.docx]

Appendix S16. 50 km global map of dominant IUCN Inland Wetland habitat classes

Ten habitat classes were present globally when the dominant class (that which covered the highest percentage) per ~50 km cell was calculated using all thematically- and empirically-derived associations. A ~50km resolution was chosen for easier viewing at the global scale. The global map of dominant classes was generated by calculating the mean percentage cover of each class across all ~1 km x 1 km cells within each 50 by 50 km cell, excluding the three un-mapped habitats (IUCN classes 5.8, 5.9, and 5.17) and subterranean habitats (IUCN class 5.18). If the maximum cover of any class per 50 km x 50 km cell was less than 0.5%, no inland wetland habitats were considered dominant in that cell. Seasonal saline, brackish, and alkaline pools (IUCN class 5.17) was dominant over the greatest extent (20,335 km^2^) and tundra wetland (IUCN class 5.10) was dominant over the lowest extent (2 km^2^, Fig 1).


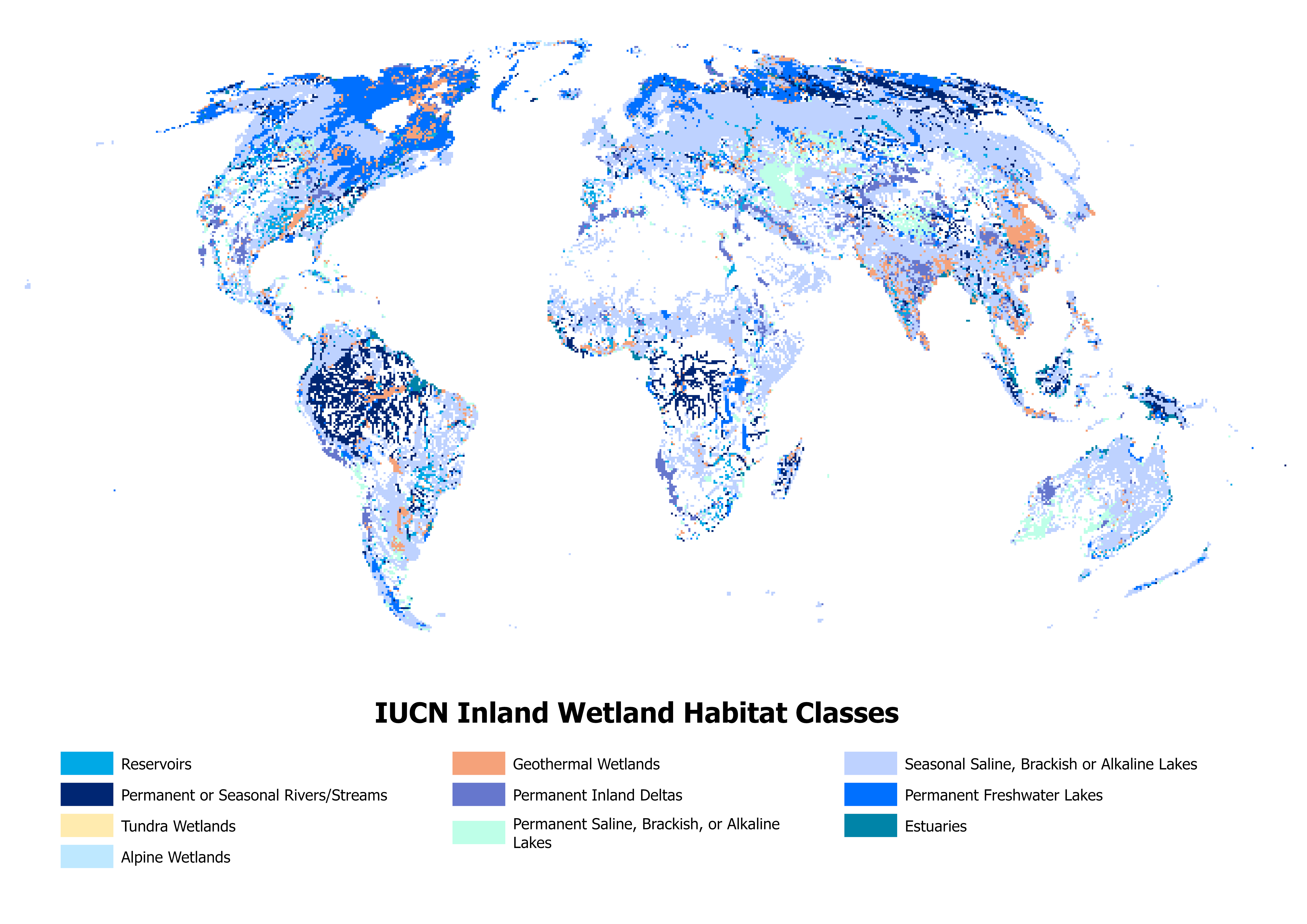


Appendix S17. The dominant wetland class per 50 km x 50 km cell globally including mapped natural inland wetlands (IUCN Habitat classes 5.1 to 5.16, excluding un-mappable classes 5.8, and 5.9), estuaries (class 9.10) and reservoirs (class 15.1). No wetland class is presented for cells where the maximum coverage of any class was less than 0.5%.
